# Comparison of Outcomes of Enucleation vs. Standard Surgical Resection for Pancreatic Neoplasms: A Systematic Review and Meta-Analysis

**DOI:** 10.3389/fsurg.2021.744316

**Published:** 2022-01-26

**Authors:** Xiaoying Shen, Xiaomao Yang

**Affiliations:** Department of Gastrointestinal Hernia Surgery, Huzhou Central Hospital, Affiliated Central Hospital Huzhou University, Huzhou, China

**Keywords:** pancreatic neoplasm, enucleation, surgical resection, meta-analysis, systematic review

## Abstract

**Background:**

With advancement in health technology, the detection rate of pancreatic neoplasms is increasing. Tissue sparing surgery (enucleation) as well as standard surgical resection are two commonly used modalities of management. There are studies comparing clinical outcomes between these two modalities; however, there is lack of studies that systematically pool the available findings to present conclusive and reliable evidence.

**Methods:**

A systematic search was conducted using the PubMed, Scopus, and Google Scholar databases. Studies that were randomised controlled trials or cohort based or analysed retrospective data were considered for inclusion. Studies should have been done in adult patients with pancreatic neoplasms and should have examined the outcomes of interest by the two management modalities i.e., enucleation and standard surgical resection. Statistical analysis was performed using STATA software.

**Results:**

A total of 20 studies were included in the meta-analysis. The operation time (in minutes) (WMD −78.20; 95% CI: −89.47, −66.93) and blood loss (in ml) (WMD −204.30; 95% CI: −281.70, −126.90) for enucleation was significantly lesser than standard surgical resection. The risk of endocrine (RR 0.32; 95% CI: 0.18, 0.56) and exocrine insufficiency (RR 0.16; 95% CI: 0.07, 0.34) was lower whereas the risk of post-operative pancreatic fistula (RR 1.46; 95% CI: 1.22, 1.75) was higher in enucleation, compared to standard surgical resection group. There were no differences in the risk of reoperation, readmission, recurrence, mortality within 90 days and 5-years overall mortality between the two groups.

**Conclusions:**

Enucleation, compared to standard surgical resection, was associated with better clinical outcomes and therefore, might be considered for selected pancreatic neoplasms. There is a need for randomised controlled trials to document the efficacy of these two management techniques.

## Introduction

Pancreatic surgeries, especially for neuroendocrine tumours, are commonly done and broadly there are two types of surgeries- tissue sparing (enucleation) and tissue resection (pancreato-duodenectomy; distal pancreatectomy) (1, 2). Pancreatic surgery is technically demanding and requires immense surgical expertise and post-operative care. Therefore, it is imperative that the nursing personnel involved in the peri as well as post-operative management are aware of the most updated evidence in terms of management of these patients. In highly specialised institutions, the rate of mortality due to pancreatic surgery/interventions is drastically reduced; however, the patients still run the risk of developing complications, particularly post-operative pancreatic fistula (POPF) and endocrine or exocrine insufficiency (3–5). Much of these side effects are due to the damage to the pancreatic parenchyma (5, 6). POPF subsequently leads to infections and post-operative pancreatic haemorrhage. These conditions increase the changes of readmission and re-operation. Tissue sparing intervention such an enucleation tends to offer some advantage over tissue resection procedures with regards to endocrine or exocrine insufficiency (5). Reduction of complications is one of the most important concerns of the surgeon and the nursing team as these ultimately increase the cost of treatment and increase the hospital stay (7, 8).

With recent increase in the technology for health imaging such as endoscopic ultrasonography, the chance of detecting smaller pancreatic lesions incidentally is increasing (9, 10). Enucleation seems to be a preferred modality for small pancreatic tumours as against standard surgical resection, particularly for those tumours that are away from the pancreatic duct (10, 11). The proportion of enucleation surgeries are increasing and therefore, there is a need to understand how this surgical modality compared with the standard surgical resection with regards to clinical outcomes. One recent systematic review by Hüttner et al. (12) compared outcomes of enucleation with standard surgical resection of pancreatic neoplasms. Using data from 22 studies, mostly observational in design, this meta-analysis found that the duration of surgery, blood loss and length of hospital stay were lower after enucleation than after standard resection. The authors also noted that the risk of post-operative endocrine and exocrine insufficiency were lower after enucleation (12). Further, the overall rate of pancreatic fistula was higher after enucleation than after standard resection. However, an important drawback of their analysis was the inclusion of several small studies with <10 patients in each arm. Such small numbers can overestimate the outcomes leading to false conclusions. Therefore, in order to present best possible evidence and update the last review the current meta-analysis was undertaken to compare outcomes of enucleation with standard surgical resection for pancreatic neoplasms by including studies with at least 10 patients per arm.

## Materials and Methods

### Search Strategy

This study was conducted according to PRISMA (Preferred Reporting Items for Systematic Reviews and Meta-analyses) guidelines. English-language publications in PubMed, Scopus and Google academic databases published prior to May 31, 2021 were systematically searched using medical topic heading (MeSH) terminology and free text words (Supplementary Table 1). This search aimed to identify studies performed on subjects with pancreatic neoplasm that compared outcomes of interest between enucleation and standard surgical resection. The primary outcomes of interest were mortality (both short and long term), risk of complications, and recurrence. Secondary outcomes were operation duration, blood loss, length of hospital stay, and risk of reoperation and readmission.

### Selection Criteria and Methods

Search results were reviewed and screened by two subject experts. Initial screening consisted of title and abstract review, with full-text review performed subsequently. Disagreements concerning study inclusion were resolved *via* discussion. Works cited by studies that met inclusion criteria were also reviewed to identify additional relevant literature.

#### Inclusion Criteria

Randomised controlled trials, cohort based trials, or retrospective data analyses were all considered for inclusion. Studies needed to examine outcomes of interest following enucleation and standard surgical resection in adult patients with pancreatic neoplasms. Studies needed to contain data on at least 10 subjects for each of the two groups to be considered for inclusion.

#### Exclusion Criteria

Case reports and review articles were excluded. Studies that did not provide data on outcomes of interest or did not provide comparative findings between enucleation and standard surgical resection were also excluded.

### Data Extraction and Quality Assessment

Two reviewers individually extracted data from included studies according to pre-determined guidelines. Data extracted included study identifiers (author information and year of study), study setting, study design, subject characteristics, overall sample size, and main findings. All included studies were assessed for quality using the Newcastle-Ottawa Quality Assessment Scale (13).

### Statistical Analysis

This meta-analysis, conducted using STATA version 16.0, reported effect sizes as pooled relative risks with 95% CIs (confidence intervals) for categorical outcomes and weighted mean differences (WMDs) for continuous outcomes. I^2^ denoted heterogeneity, and where *I*^2^ exceeded 50%, a random effects model was used (14). A *p*-value of < 0.05 indicated statistical significance. Egger's test was employed to assess publication bias.

## Results

### Study Selection, Characteristics, and Quality

**Figure 1:**
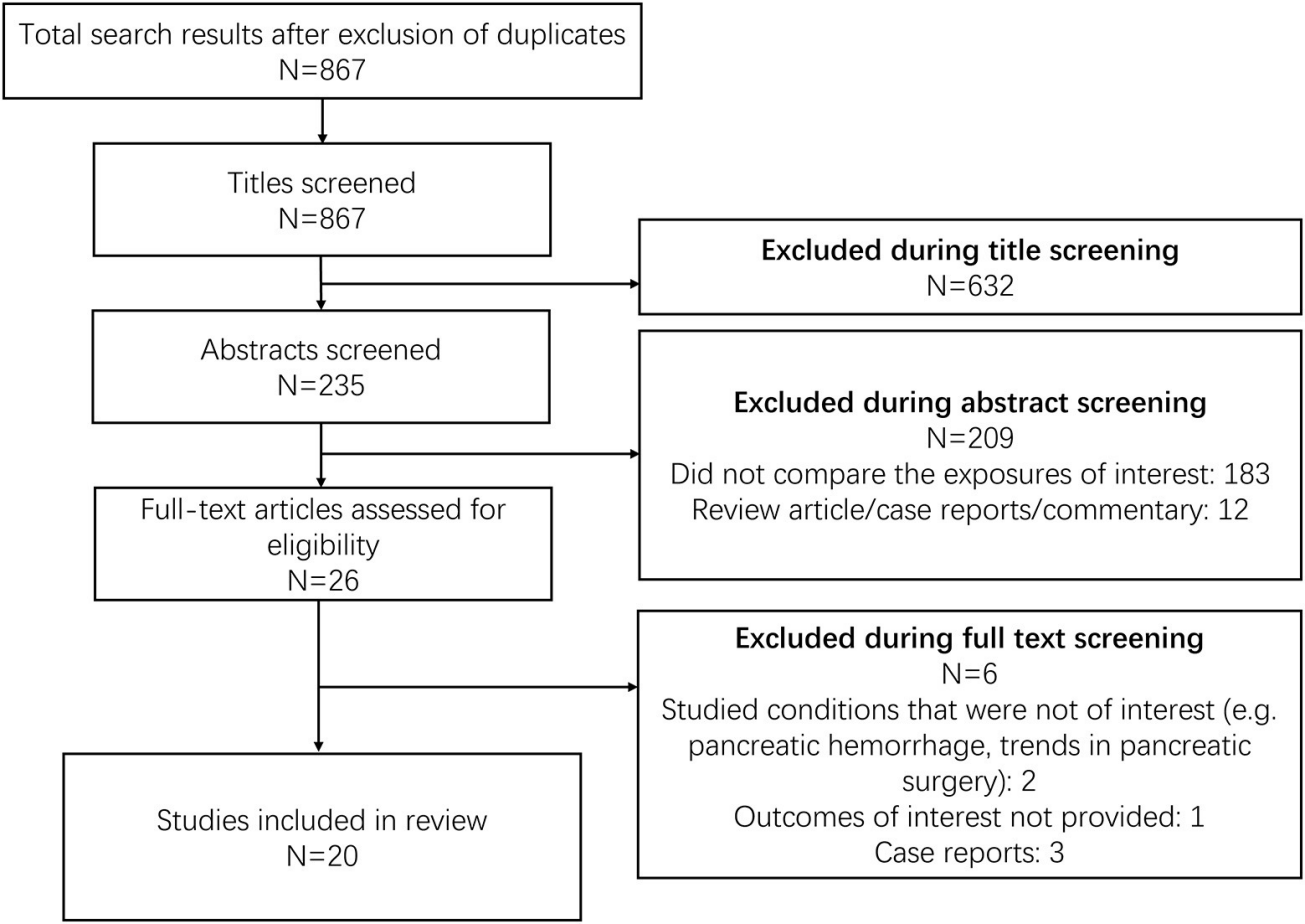
Selection process of the studies included in the review.

The search strategy obtained 867 unique study candidates (Figure 1). Title screening reduced the number of eligible candidates to 235, and abstract screening further reduced this number to 26. These 26 papers underwent detailed full-text review, with 20 meeting inclusion criteria (Supplementary Table 2) (10, 15–33). Two studies were prospective non-randomised in design and one was a case-control study. The remaining 17 were retrospective data based. Six studies were done in the USA, five in China, two each in Germany, Norway, and Italy, and one each in the Netherlands, Spain, and France. In almost all the included studies, majority of the participants had tumour located either in the body or tail of the pancreas, except for the study by Hackert et al. where more than 50% of the subjects had tumour in the head/uncinate process (27). All included studies had a follow up period of at least 18 months, and were of good quality (Supplementary Tables 2, 3).

### Operation Duration, Blood Loss, and Length of Hospital Stay

**Figure 2:**
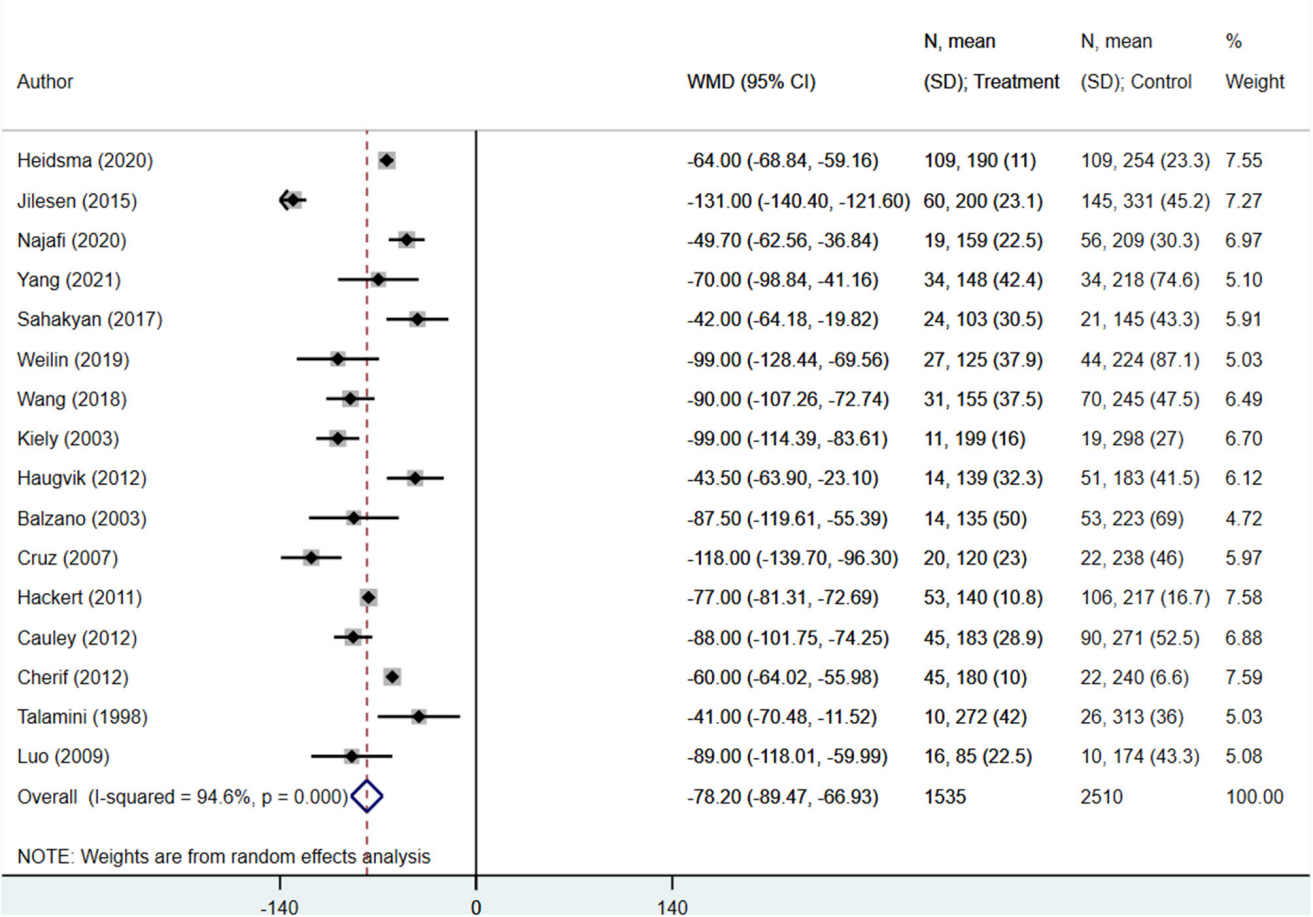
Comparison of operation time (in minutes) between enucleation and standard surgical resection.

**Figure 3:**
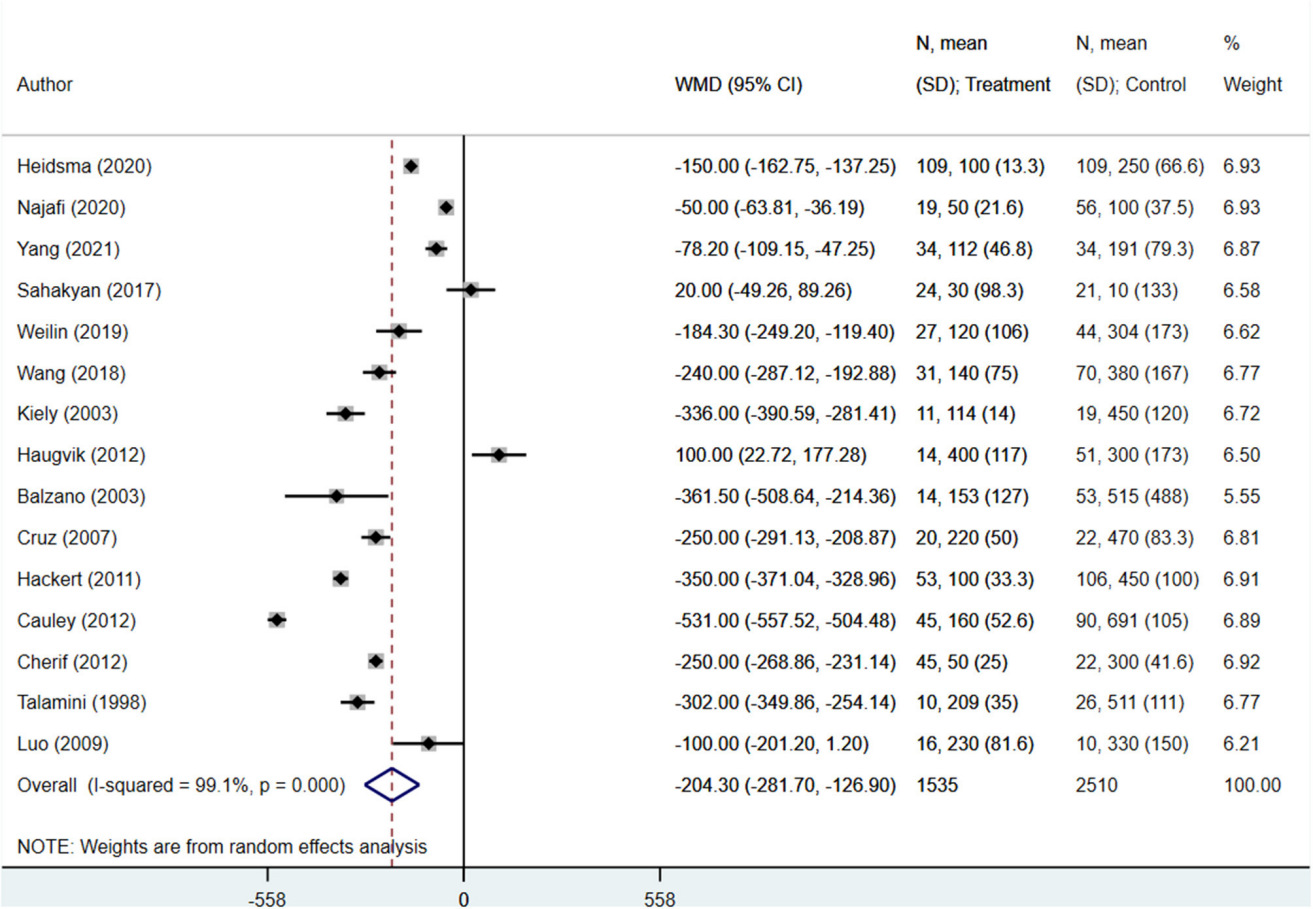
Comparison of blood loss (in ml) between enucleation and standard surgical resection.

**Figure 4:**
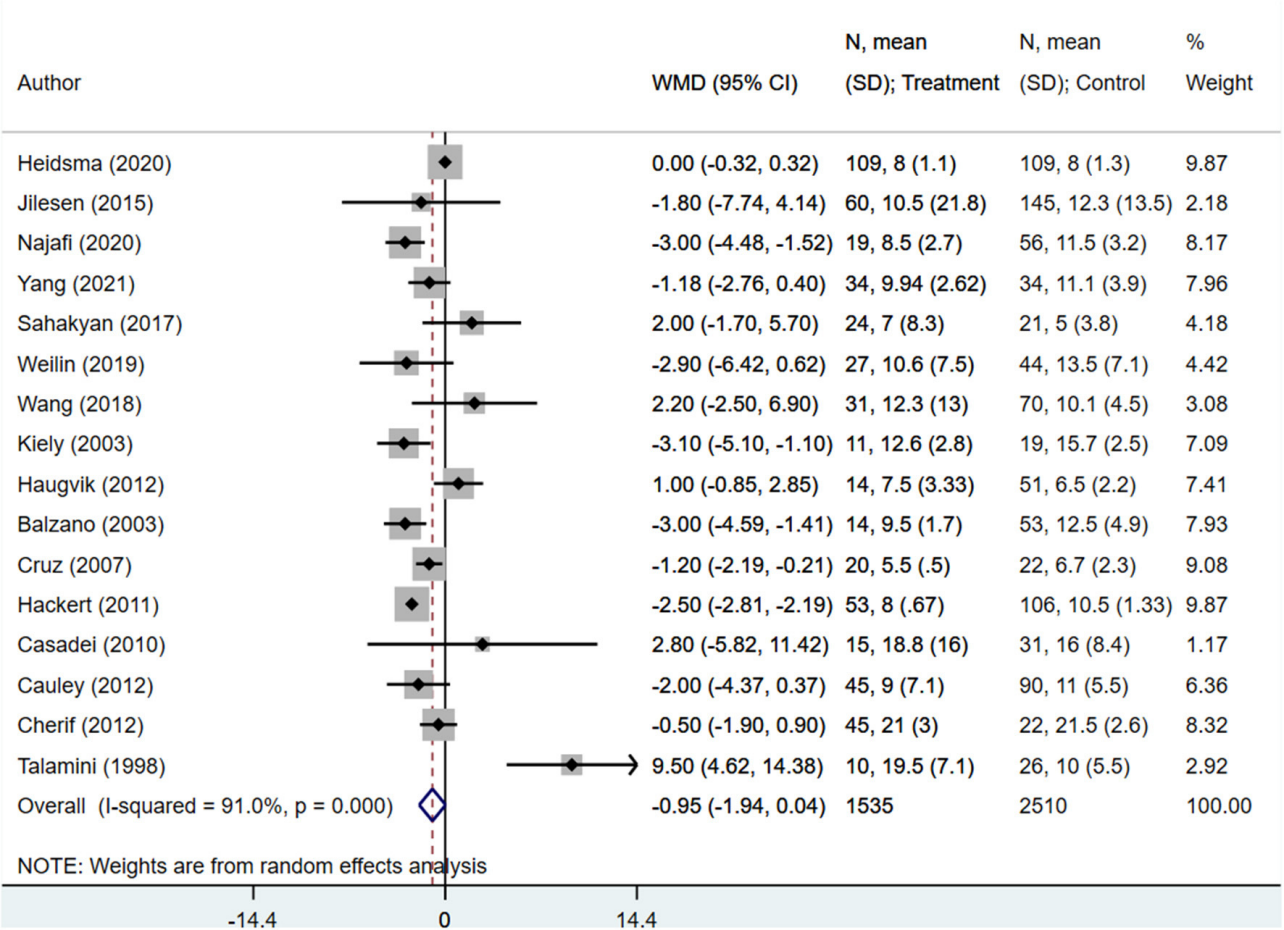
Comparison of length of hospital stay (in days) between enucleation and standard surgical resection.

The operation duration (in minutes) for enucleation patients was significantly shorter than those undergoing standard surgical resection (WMD −78.20; 95% CI: −89.47, −66.93; *I*^2^ = 94.6%, *N* = 16) (Figure 2). Similarly, patients undergoing enucleation also showed lower blood loss (in ml) (WMD −204.30; 95% CI: −281.70, −126.90; *I*^2^ = 99.1%, *N* = 15) (Figure 3). Finally, hospital stays (in days) were shorter for patients in the enucleation group, although the difference only trended towards statistical significance (WMD −0.95; 95% CI: −1.94, 0.04; *I*^2^ = 91.0%, *N* = 16) (Figure 4). Egger's test did not indicate the presence of publication bias (*P* = 0.33 for operation duration, *P* = 0.24 for blood loss, and *P* = 0.71 for length of hospital stay).

### Reoperation, Readmission, and Endocrine and Exocrine Insufficiency

**Figure 5:**
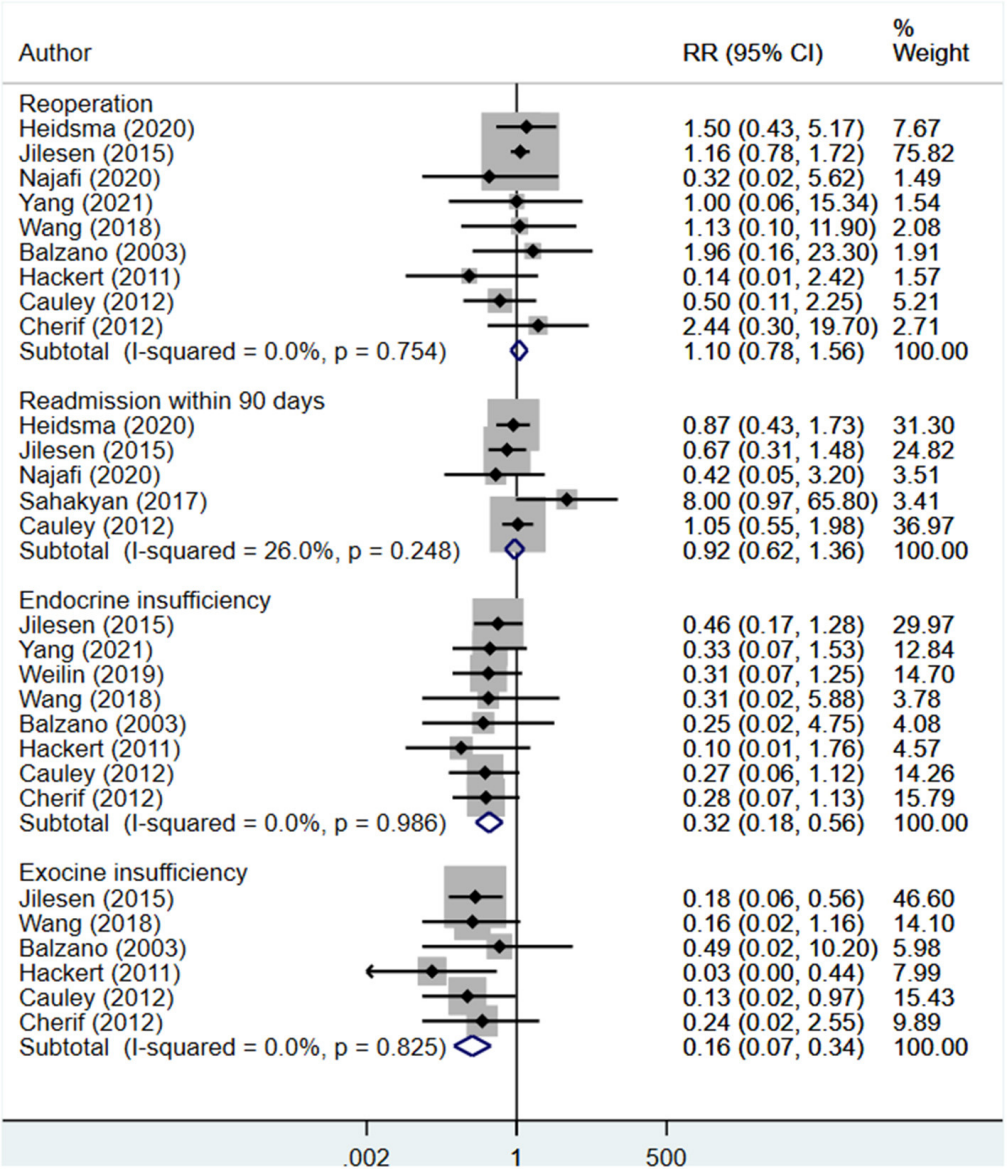
Comparison of risk of reoperation, readmission, endocrine insufficiency and exocrine insufficiency between enucleation and standard surgical resection.

The risk of reoperation (RR 1.10; 95% CI: 0.78, 1.56; *I*^2^ = 0.0%, *N* = 9) and readmission (RR 0.92; 95% CI: 0.62, 1.36; *I*^2^ = 26.0%, *N* = 5) within 90 days were both similar in both groups (Figure 5). However, the risks of endocrine (RR 0.32; 95% CI: 0.18, 0.56; *I*^2^ = 0.0%, *N* = 8) and exocrine insufficiency (RR 0.16; 95% CI: 0.07, 0.34; *I*^2^ = 0.0%, *N* = 6) were lower in enucleation patients compared to the standard surgical resection group (Figure 5). Egger's test did not indicate the presence of publication bias (*P* = 0.29 for reoperation, *P* = 0.18 for readmission, *P* = 0.27 for endocrine insufficiency, and *P* = 0.53 for exocrine insufficiency).

### Complications, Recurrence, and Survival

**Figure 6:**
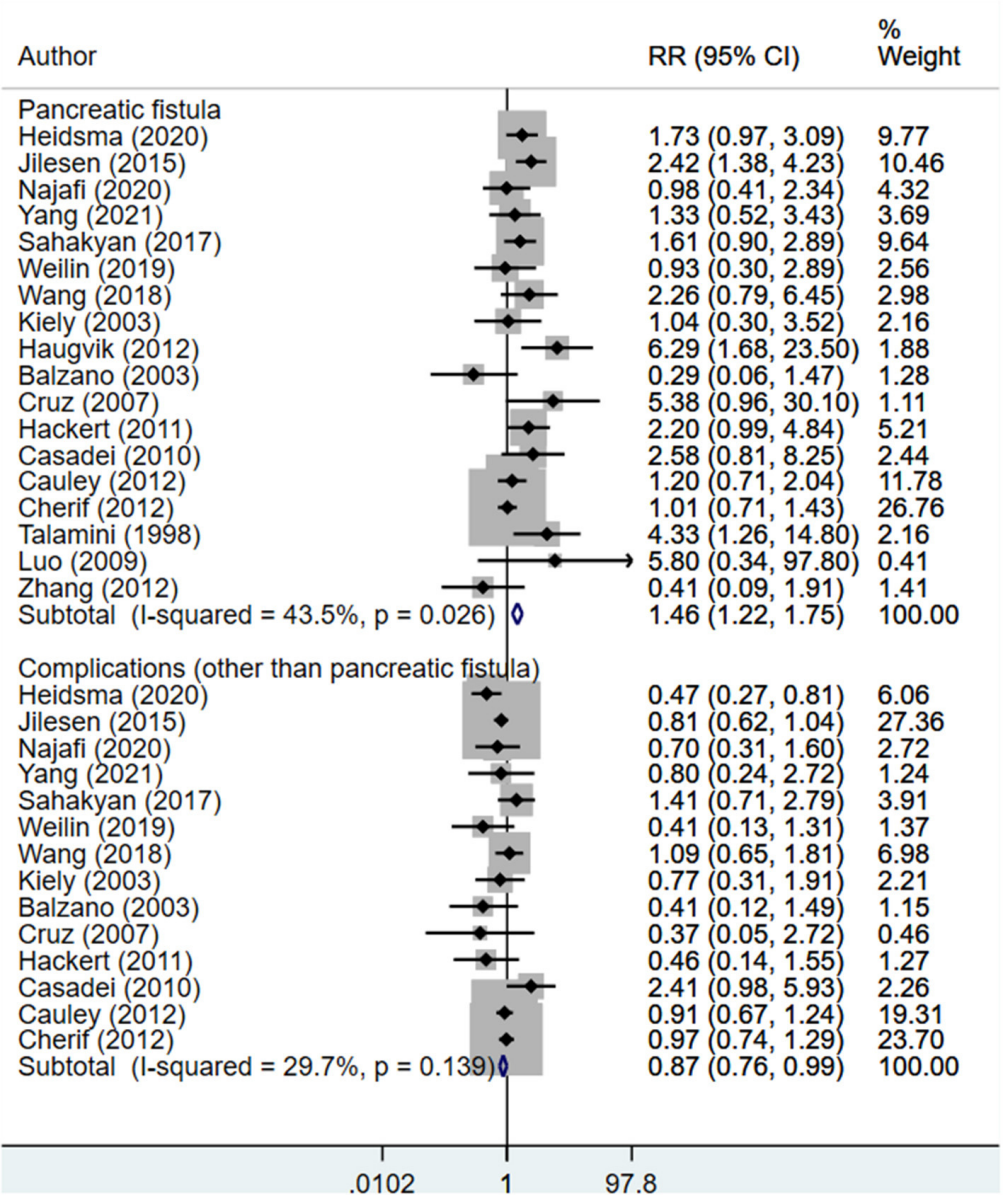
Comparison of risk of complications between enucleation and standard surgical resection.

**Figure 7:**
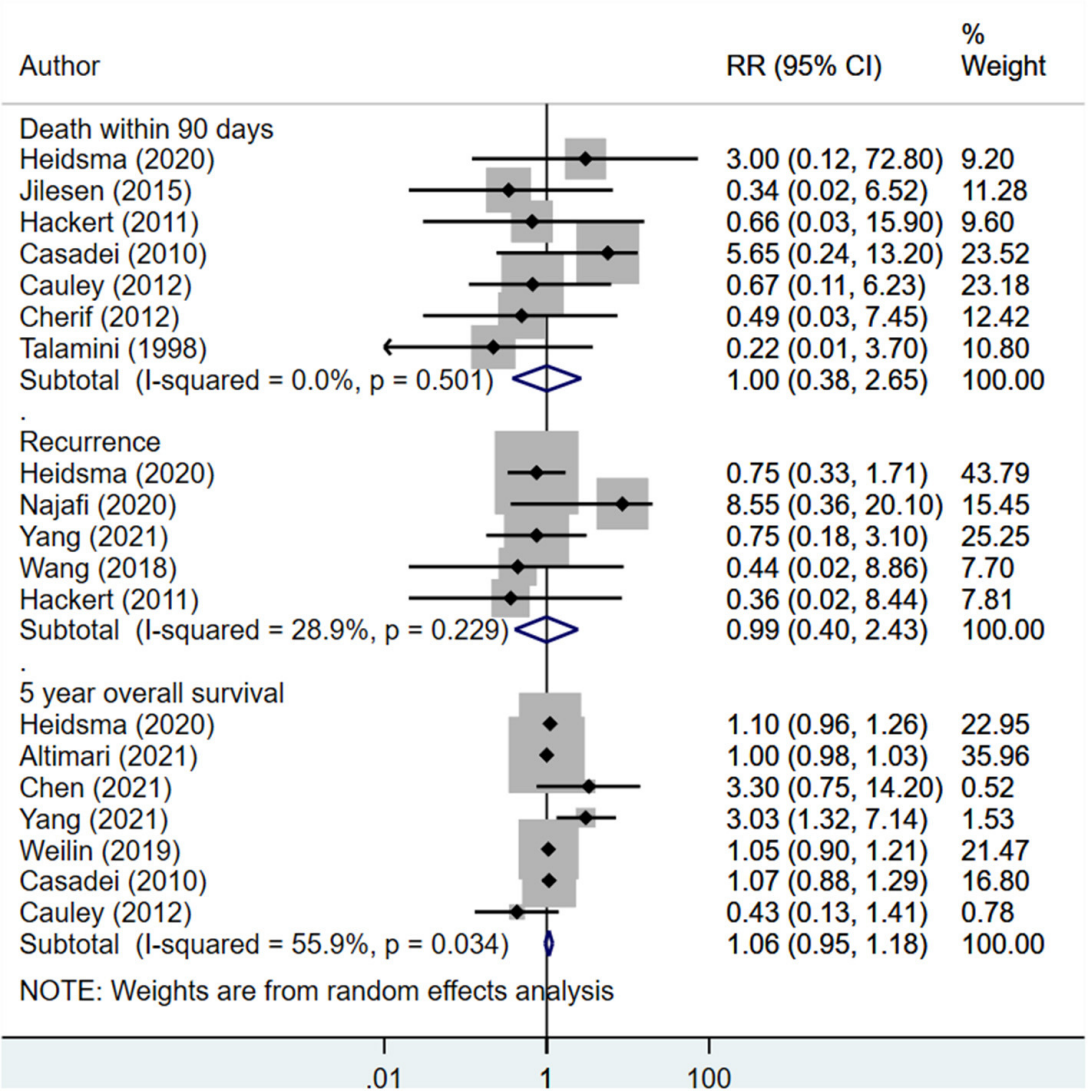
Comparison of risk of recurrence, death within 90 days of operation and 5-years overall survival between enucleation and standard surgical resection.

The risk of post-operative pancreatic fistula was higher in those undergoing enucleation compared to standard surgical resection (RR 1.46; 95% CI: 1.22, 1.75; *I*^2^ = 43.5%, *N* = 18). However, the risk of other complications was lower in the enucleation group (RR 0.87; 95% CI: 0.76, 0.99; *I*^2^ = 29.7%, *N* = 14) (Figure 6). Egger's test did not indicate the presence of publication bias (*P* = 0.81 for post-operative pancreatic fistula, *P* = 0.33 for other complications). The risk of recurrence (RR 0.99; 95% CI: 0.40, 2.43; *I*^2^ = 28.9%, *N* = 5), rate of death within 90 days of operation (RR 1.00; 95% CI: 0.38, 2.65; *I*^2^ = 0.0%, *N* = 7), and 5-year overall survival (RR 1.06; 95% CI: 0.95, 1.18; *I*^2^ = 55.9%, *N* = 7) were all similar between both groups (Figure 7). Egger's test did not indicate the presence of publication bias (*P* = 0.70 for recurrence, *P* = 0.12 for death within 90 days, and *P* = 0.39 for 5-years overall survival).

## Discussion

The current meta-analysis was conducted to evaluate the two treatment modalities for management of pancreatic tumours with respect to risk of mortality, complications (including endocrine and exocrine insufficiency) and recurrence. Other outcomes of interest were operation time, blood loss, length of hospital stay, risk of reoperation and readmission. The key findings of the meta-analysis were that enucleation was associated with lower operative time, blood loss and hospital stay but a higher risk of post-operative pancreatic fistula. Enucleation was associated with lower risk of endocrine as well as exocrine insufficiency. There were no differences in the two groups based on risk of readmission, reoperation, recurrence and overall mortality (both short and long term).

The findings support the earlier meta-analysis by Hüttner et al. where similar findings were reported (12). Hüttner et al. found, through pooling of 22 studies (*N* = 1,148 patients), documented the superiority of pancreatic enucleation with respect to reduced intraoperative blood loss, reduced blood loss and duration of hospital stay. Similar to our review, the earlier meta-analysis found reduced risk of endocrine as well as exocrine insufficiency along with increased risk of POPF in subjects undergoing enucleation compared to standard surgical resection (12).

Of particular interest are the findings related to lower duration of surgery, blood loss and hospital stay. All these three parameters contribute substantially towards the health care costs, particularly in surgical units (34, 35). Enucleation, therefore, could be viewed as a more cost-effective treatment modality from the perspective of a health care manager. It should be noted that enucleation should not be considered as a simple surgical procedure as the risk of pancreatic fistula is substantial. Post-operative pancreatic fistula (POPF) is a major problem for patients undergoing pancreatic surgery and efforts are made to decrease POPF, not only to decrease the costs for the health care system, but more importantly to improve the patients' quality of life. Fast recovery after surgery is crucial for the much-needed adjuvant systemic therapy, which could potentially affect the overall survival. The increased risk of POPF after enucleation is concerning in this regard. We carefully evaluated the included studies to examine the operational definition of post-operative pancreatic fistula used to rule out the possibility that pancreatic fistula might be defined as biochemical leak which usually does not impact surgical outcomes in patients. We noted that majority of the included studies defined pancreatic fistula using the International Study Group Definition i.e., any measurable drainage from an operatively placed drain on or after post-operative day 3, with amylase content >3 times the upper limit of normal serum amylase levels. Some studies defined POPF as drainage of more than 50 ml of amylase-rich fluid through operatively placed drains on or after post-operative day 7 and some as the drain output from the peripancreatic drainage exceeding 10 ml/day with an amylase level 3 times higher than the serum value on or after post-operative day 7.

With the advent of laparoscopic surgery and its wide availability at many health centres, there is a need for more evidence to understand how enucleation performs in comparison to the standard surgical resection. Enucleation is usually reserved for benign or low-grade tumours that are at a distance from the pancreatic duct (36, 37). The technique requires high level of technicality and skill but the advantage is that it preserves pancreatic parenchyma and consequently, pancreatic tissue and function. It requires a careful examination to assess which lesions could be considered for enucleation and also highly skilled surgeons would be better placed to do this procedure in a setup that has advanced health care facilities.

One of the interesting findings of the meta-analysis by Hüttner et al. was that specialised centres often lead to lower complication rates both for enucleation and surgical resection (12). Nurses are an integral part of the team in the management of patients with pancreatic neoplasm. They bring a wide array of skills and help in the implementation of the treatment plan. It is therefore necessary that the nursing team has access to the most updated evidence on the management modalities for pancreatic tumours so that they can contribute to the multidisciplinary health care team. The findings of the present meta-analysis would be useful to the pancreatic oncology nursing team as it presents the most updated comparative evidence on the clinical efficacy between enucleation and surgical resection.

There are some limitations of the current meta-analysis that was conducted using studies with limited number of subjects. One of the limitations is the availability of non-randomised studies on the issue under consideration. Therefore, the findings emerging from this meta-analysis should be interpreted with caution as the possibility of bias cannot be ruled out. As the included studies were retrospective data based, the possibility of non-adjustment for potential confounders could not be ruled out. There is a need for robust randomised controlled trials to effectively test the outcomes among these two management modalities. However, conducting such a trial may be difficult due to the rarity of pancreatic lesions amenable to either enucleation or standard surgical resection. Further, as the incidence of pancreatic cancers are relatively lower, a trial would need a large sample size. Another limitation is the scarcity of studies reporting on long term outcomes such as recurrence free survival or overall survival. Only 7 out of the included 20 studies reported on 5-years overall survival in the present meta-analysis. Most of the studies focussed on short-term outcomes. While several studies offer evidence that the disease-free survival after enucleation and standard resections in patients with pancreatic neuroendocrine tumours might be comparable, the present meta-analysis does not provide conclusive answer to this. An important limitation of the current meta-analysis is that we were unable to compare enucleation vs. standard resection according to the site of the tumour (head vs. body-tail of the pancreas). This is because in almost all the included studies, most of the subjects had tumour located in the body or tail of pancreas, with the exception of one study by Hackert et al. where >50% of the subjects had tumour in the head/uncinate of the pancreas. A pancreaticoduodenectomy for a tumour in the head of the pancreas has very different risk for surgical complications than an enucleation of the same tumour in the body-tail. Stratified pooling of findings, based on the location of the tumour, was not possible as in all the included studies, the authors provided overall findings and not separately as per the tumour site i.e., body/tail or head of the pancreas.

Another limitation is that we could not undertake stratified analysis based on the nature of pancreatic pathology. While most of the studies included patients with pancreatic neuroendocrine tumours (pNETs), there were also studies that had subjects with a more heterogenous pathology i.e., those with pNETs, intraductal papillary mucinous neoplasm (IPMN) and solid pseudopapillary neoplasm (SPN). Different pathologies carry different risks for recurrence, mortality and post-operative fistula. Studies also included a heterogenous population in terms of the tumour size. The size of the tumour is also associated with risk for lymph node metastases and tumour aggressiveness. It would have been better to conduct a subgroup analysis based on tumour pathology and size but due to lack of such stratified findings in the included studies, such an analysis could not be undertaken.

## Conclusions

The current meta-analysis, using studies that were retrospective in design, found that enucleation, compared to standard surgical resection was associated with better clinical outcomes such as reduced operative time, blood loss, length of hospital stay as well as reduced risk of endocrine and exocrine insufficiency. However, enucleation was associated with increased risk of post-operative pancreatic fistula. There is a need for randomised controlled trials, probably multicentric in nature, to effectively document the efficacy of these two management techniques. Careful interpretation of the findings should be done as selected cohort of subjects in majority of the included studies was heterogeneous, mainly with respect to tumour pathology, size and location.

## Data Availability Statement

The original contributions presented in the study are included in the article/Supplementary Material, further inquiries can be directed to the corresponding author.

## Author Contributions

XS and XY conceived and designed the study. XS was involved in literature search, data collection, and wrote the paper. XY analysed the data and reviewed and edited the manuscript. All authors read and approved the final manuscript.

## Conflict of Interest

The authors declare that the research was conducted in the absence of any commercial or financial relationships that could be construed as a potential conflict of interest.

## Publisher's Note

All claims expressed in this article are solely those of the authors and do not necessarily represent those of their affiliated organizations, or those of the publisher, the editors and the reviewers. Any product that may be evaluated in this article, or claim that may be made by its manufacturer, is not guaranteed or endorsed by the publisher.
