# Supplementary material for: Comparison of Outcomes of Enucleation vs. Standard Surgical Resection for Pancreatic Neoplasms: A Systematic Review and Meta-Analysis
Source: Front Surg. 2022 Jan 26;8:744316. doi: 10.3389/fsurg.2021.744316 (PMC8825491; doi:10.3389/fsurg.2021.744316)
Supplement: Supplementary file 1 [file Table_1.docx]

**Supplementary table 1. Characteristics of the studies included in the meta-analysis**

| **Author (year of publication)** | **Study design** | **Country** | **Participant characteristics** | **Comparison groups** | **Sample size** | **Key outcome (enucleation compared to standard surgical resection)** |
| --- | --- | --- | --- | --- | --- | --- |
| Heidsma et al. (2020) [15] | Retrospective study | USA | Patients with pancreatic neuroendocrine tumor (pNET); majority males (46%); Mean age: 54 yrs; majority of the tumours were located in body or tail (67%); majority were low grade (G1) early-stage tumours (T1; Nx or N0) (>65%); median tumour size (in mm): 21 mm | enucleation (EN) vs. Pancreato-duodenectomy (PD) or distal pancreatectomy (DP) | 218 (enucleation: 109; surgical resection: 109) | *Median follow-up of 47 months* (*EN patient), 37 months (PD/DP patients)*  **Operating time (mean, SD), min:** 190 (11) vs. 254 (23.3)  **Estimated blood loss (mean, SD), mL:** 100 (13.3) vs. 250 (66.6)  **Complications (other than pancreatic fistula):** RR 0.47 (95% CI: 0.27, 0.81)  **Postoperative pancreatic fistula:** RR 1.73 (95% CI: 0.97, 3.09)  **Length of hospital stay, (Mean, SD) days:** 8 (1.1) vs. 8 (1.3)  **Need for reoperation:** RR 1.5 (95% CI: 0.43, 5.17)  **Readmission <90 days:** RR 0.87 (95% CI 0.43, 1.73)  **Death, <90 days:** RR 3.0 (95% CI: 0.12, 72.8)  **Recurrence:** RR 0.75 (0.33, 1.71)  **5-year survival**: RR 1.10 (95% CI: 0.96, 1.26) |
| Jilesen et al. (2015) [16] | Retrospective study | Netherlands, | Mean age of 50 years; 55% were females; 63% had non-functional tumour. Median tumor size (in mm): 13 mm; majority of the tumours were in the body and tail (83%) | Pancreatic enucleation vs. resection—pancreato-duodenectomy | 205 (Tumor enucleation- 60; Pancreatectomy: 145) | *Median follow-up time of 29 months*  **Mean operative time (in minutes):** 200 (23.1) vs. 331 (45.2)  **Complications (other than pancreatic fistula):** RR 0.81 (95% CI: 0.62, 1.04)  **Length of hospital stay, days:** 10.5 (21.8) vs. 12.3 (13.5)  **Postoperative pancreatic fistula:** RR 2.42 (95% CI: 1.38, 4.23)  **Readmission within 30 days**: RR 0.67 (95% CI: 0.31, 1.48)  **Need for reoperation:** RR 1.16 (95% CI: 0.78, 1.72)  **In-hospital mortality:** RR 0.34 (95% CI: 0.02, 6.52)  **Endocrine insufficiency:** RR 0.46 (95% CI: 0.17, 1.28)  **Exocrine insufficiency:** RR 0.18 (95% CI: 0.06, 0.56) |
| Altimari et al. 2021 [17] | Retrospective study | USA | Patients with diagnosis of pNET who underwent surgical resection were included; Age, Median (IQR): 60 (17); 48.57% were males; 57% had tumor in body or tail; Median tumor size was 15 mm | Enucleation vs. Surgical resection | 4083 (Oncologic Resection: 3086; Enucleation: 997) | *Median follow up 40 months for both the groups*  **5-year survival rate**: RR 1.00 (95% CI: 0.98, 1.03) |
| Chen et al. 2021 [18] | Retrospective study | USA | Patients with diagnosis of pNET who underwent radical or non-radical resection were included; 51.8% were Males; 57.8% had tumor in the body or tail of pancreas; Non-functional tumor: 92.2%; No Distant metastasis: 85.4%; Majority with tumor size ≤40 mm | Enucleation vs. Surgical resection | 1387 (Enucleation: 129; standard surgical resection: 1258) | *Median follow-up time 37 months*  **5- year Cancer specific survival:** RR 3.2 (95% CI: 0.40, 26.16)  **5- year overall survival:** RR 3.3 (95% CI: 0.75, 14.2) |
| Najafi et al. 2020 [19] | Retrospective study | Germany | Median age: 53 years; >50% were females; 95% had benign and borderline tumor; Median tumor size 17 mm; >95% had tumor location in the body/tail. | Enucleation vs. distal pancreatic resection | 75 (Enucleation: 19, Distal pancreatic resection: 56) | *Median follow up 58 months*  **Complication (other than pancreatic fistula):** RR 0.70 (95% CI: 0.31, 1.60)  **Postoperative pancreatic fistula:** RR 0.98 (95% CI: 0.41, 2.34)  **90-Day readmission:** RR 0.42 (95% CI: 0.05, 3.20)  **Local recurrence (glucagonoma**): 8.55 (0.36, 20.1)  **Need for reoperation:** RR 0.32 (95% CI: 0.02, 5.62)  **Operating time (mean, SD), min:** 159.25 (22.5) vs. 209 (30.3)  **Estimated blood loss (mean, SD), mL:** 50 (21.6) vs. 100 (37.5)  **Length of hospital stay, days:** 8.5 (2.7) vs. 11.5 (3.2)  **No mortality in each group** |
| Yang et al. 2021 [20] | Retrospective study | China | Females: 69.7%; Mean age: 51.07; Mean tumor size, mm: 17; 73% had tumor body/tail; Majority with WHO G1 classification and AJCC stage I/II | Enucleation and regular pancreatectomy (RP) | 68 (Enucleation, EN: 34; Regular pancreatectomy, RP: 34) | *Median follow-up period 60.76 months in the EN group and 43.29 months in the RP group*  **Surgical duration (min):** 147.94 (42.39) vs. 217.94 (74.60)  **Estimated blood loss (ml):** 112.35 (46.84) vs. 190.59 (79.31)  **Postoperative complications (other than pancreatic fistula):** RR 0.80 (95% CI: 0.24, 2.72)  **Postoperative pancreatic fistula:** RR 1.33 (95% CI: 0.52, 3.43)  **Re-operation:** RR 1.0 (95% CI: 0.06, 15.34)  **Endocrine insufficiency:** RR 0.33 (95% CI: 0.07, 1.53)  **Length of hospital stay, days:** 9.94 (2.62) vs. 11.12 (3.90)  **5- year disease free survival:** RR 2.8 (95% CI: 1.37, 5.55)  **5-year overall survival:** RR 3.03 (95% CI: 1.32, 7.14)  **Tumour recurrence:** RR 0.75 (95% CI: 0.18, 3.10) |
| Sahakyan et al. 2017 [21] | Case series | Norway | Mean age was around 50 years; Over 60% were Females; Mean tumor size was around 1.86 (1); | Case-matched  comparison between the  patients undergoing enucleation and distal pancreatectomy | 45 (laparoscopic pancreatic enucleation: 24; pancreatectomy: 21) | *Median follow-up time was 18 months*  **Operating time (mean, SD), min:** 103 (30.5) vs. 145 (43.3)  **Estimated blood loss (mean, SD), mL:** 30 (98.3) vs. 10 (133.3)  **Complications (other than pancreatic fistula):** RR 1.41 (95% CI 0.71, 2.79)  **Postoperative pancreatic fistula:** RR 1.61 (95% CI: 0.90, 2.89)  **Length of hospital stay (mean, SD), days:** 7 (8.3) vs. 5 (3.8)  **Readmission:** RR 8 (95% CI: 0.97, 65.82) |
| Weilin et al. 2019 [22] | Retrospective study | China | Majority were males and aged >50 years; Mean tumor diameter: 1.44 (0.37); around 63% tumour in body/tail | Enucleation versus regular pancreatectomy | 71 (Enucleation: 27; regular pancreatectomy: 44) | *Median follow-up time was 77 months*  **Surgical duration(min):** 125.33 (37.970) vs. 224.34 (87.144)  **Estimated blood loss (ml):** 120 (106.2) vs. 304.32 (172.8)  **Postoperative length of hospital stay:** 10.56 (7.475) vs. 13.45 (7.086)  **Postoperative pancreatic fistula:** RR 0.93 (95% CI: 0.30, 2.89)  **Complications (other than pancreatic fistula):** RR 0.41 (95% CI: 0.13, 1.31)  **Endocrine insufficiency:** RR 0.31 (95% CI: 0.07, 1.25)  **Overall 5-year survival:** RR 1.05 (95% CI: 0.90, 1.21) |
| Wang et al. 2018 [23] | Retrospective study | China | Median age of around 30 years; >80.0% were females; median tumor size: 55 mm; in 50% of the subjects, tumor located in body/tail | Enucleation (EN group)  vs. Conventional pancreatic surgery (non-EN) | 101 (EN: 31; conventional pancreatic resection: 70) | *Mean follow-up length was 46.1 months for EN group, 42.2 months for Non-EN group*  **Operation time (min):** 155 (37.5) vs. 245 (47.5)  **Intraoperative blood loss (ml):** 140 (75) vs. 380 (166.6)  **Complications (other than pancreatic fistula):** RR 1.09 (95% CI: 0.65, 1.81)  **Postoperative pancreatic fistula**: RR 2.26 (95% CI: 0.79, 6.45)  **Hospital stay (Mean SD, day):** 12.3 (13) vs. 10.1 (4.5)  **Reoperation:** RR 1.13 (95% C: 0.10, 11.99)  **Recurrence:** RR 0.44 (95% CI: 0.02, 8.86)  **Endocrine insufficiency**: RR 0.31 (95% CI: 0.02, 5.88)  **Exocrine insufficiency:** RR 0.16 (95% CI: 0.02, 1.16) |
| Kiely et al. 2003 [10] | Retrospective study | USA | Mean age: 56 years; 73% were females; 63% were asymptomatic; mean size of tumour around 37mm; 57% of the tumour in body/tail; majority were mucinous cystic neoplasm or serous cyst adenoma | Enucleation vs. Resection | 30 (Enucleation: 11; Resection: 19) | *Mean follow-up time for all patients was 38.4*  *months*  **Operative time (Mean (SD), min):** 199 (16) vs. 298 (27)  **Blood loss (Mean (SD), mL):** 114 (14) vs. 450 (120)  **Pancreatic fistulas**: RR 1.04 (95% CI: 0.30, 3.52)  **Complication (other than pancreatic fistula):** RR 0.77 (95% CI: 0.31, 1.91)  **Postoperative length of stay (Days):** 12.6 (2.8) vs. 15.7 (2.5)  None of the patients in either of the groups had recurrence, mortality, endocrine or exocrine insufficiency. |
| Haugvik et al. 2012 [24] | Retrospective study | Norway | Median age: 59 years; 52% were Females; Median BMI of 26 kg/m^2^; in majority (n=52), tumour location was in body/tail of pancreas; majority has pancreatic neuroendocrine tumour-insulinoma | Enucleation vs. laparoscopic surgery | 65 (Enucleation: 14; Resection: 51) | *Mean follow-up time was 51 months*  **Post-operative pancreatic fistula:** RR 6.29 (95% CI: 1.68, 23.45)  **Mean (SD) operative time (minutes):** 139 (32.3) vs. 182.5 (41.5)  **Mean (SD) intraoperative blood loss (ml):** 400 (116.6) vs. 300 (172.5)  **Mean (SD) length of hospital stay (days):** 7.5 (3.33) vs. 6.5 (2.16)  No mortality was observed |
| Balzano et al. 2003 [25] | Retrospective study | Italy | Mean age: 53 years; 63% were Females; mostly benign histology (endocrine tumour followed by cystadenoma) | Enucleation vs. laparoscopic surgical resection | 67 (Enucleation: 14; Resection: 53) | *Mean follow-up time was 66 months*  **Post-operative pancreatic fistula:** RR 0.29 (95% CI: 0.06, 1.47)  **Mean (SD) operative time (minutes):** 135 (50) vs. 222.5 (69)  **Mean (SD) intraoperative blood loss (ml):** 153 (127) vs. 514.5 (487.5)  **Mean (SD) length of hospital stay (days):** 9.5 (1.7) vs. 12.5 (4.95)  **Reoperation:** RR 1.96 (95% CI: 0.16, 23.3)  **Complication (other than pancreatic fistula):** RR 0.41 (95% CI: 0.12, 1.49)  **Endocrine insufficiency**: RR 0.25 (95% CI: 0.02, 4.75)  **Exocrine insufficiency:** RR 0.49 (95% CI: 0.02, 10.2)  No mortality was observed |
| Cruz et al. 2007 [26] | Retrospective study | Spain | Mean age of around 57 yrs; majority were females; median tumour size of around 53mm; majority of subjects with tumour in the body/tail | Enucleation vs. laparoscopic surgical resection (distal pancreactomy) | 42 (Enucleation: 20; Resection: 22) | *Mean follow up of >36 months*  **Mean (SD) operative time (minutes):** 120 (23) vs. 238 (46)  **Mean (SD) intraoperative blood loss (ml):** 220 (50) vs. 470 (83.3)  **Post-operative pancreatic fistula:** RR 5.38 (95% CI: 0.96, 30.1)  **Complication (other than pancreatic fistula):** RR 0.37 (95% CI: 0.05, 2.72)  **Mean (SD) length of hospital stay (days):** 5.5 (0.5) vs. 6.7 (2.3) |
| Hackert et al. 2011 [27] | Prospective study | Germany | Majority females (>60%); Median age: 55 years; Median tumor size of <30 mm; majority (>50%) had Neuroendocrine tumor; majority (>50%) had tumor in Head/uncinate process | Enucleation vs. Standard resections | 159 (Enucleation: 53; Standard resections: 106) | *Median follow-up was 27 months*  **Post-operative pancreatic fistula:** RR 2.20 (95% CI: 0.99, 4.84)  **Mean (SD) operative time (minutes):** 140 (10.8) vs. 217 (16.7)  **Mean (SD) intraoperative blood loss (ml):** 100 (33.3) vs. 450 (100)  **Mean (SD) length of hospital stay (days):** 8.0 (0.67) vs. 10.5 (1.33)  **Reoperation:** RR 0.14 (95% CI: 0.01, 2.42)  **Recurrence:** RR 0.36 (95% CI: 0.02, 8.44)  **Complication (other than pancreatic fistula):** RR 0.46 (95% CI: 0.14, 1.55)  **Endocrine insufficiency**: RR 0.10 (95% CI: 0.01, 1.76)  **Exocrine insufficiency:** RR 0.03 (95% CI: 0.002, 0.44)  **Mortality:** RR 0.66 (95% CI: 0.03, 15.9) |
| Casadei et al. 2010 [28] | Prospective study | Italy | Female: 58.7%; Mean age: 55.2 yrs; Size of tumor, mean: 20 mm; around 70% had tumor in body/tail; 52% had non-functioning hormonal tumour | Enucleation vs. standard surgical resection | 46 (Enucleation: 15; surgical resection: 31) | *Mean follow up 10 years in EN group and 8.4 years in Typical Resection*  **Postoperative mortality (Death):** RR 5.65 (95% CI: 0.24, 13.2)  **Postoperative complications (other than pancreatic fistula)**: RR 2.41 (95% CI: 0.98, 5.93)  **Pancreatic fistula:** RR 2.58 (95% CI: 0.81, 8.25)  **Postoperative hospital stay (Mean, SD, days):** 18.8 (16) vs. 16 (8.4)  **5-year survival:** RR 1.07 (95% CI:0.88, 1.29) |
| Cauley et al. 2012 [29] | Retrospective study | USA | Mean age of around 53 years; Nearly 60% were Females; >50% had tumor location in body/tail; Tumor size: around 25mm | Enucleation vs. Resection | 135 (Enucleation: 45; Resection: 90) | **Mean (SD), Operative time (min):** 183 (28.9) vs. 271 (52.5)  **Mean (SD), Blood loss (mL):** 160 (52.6) vs. 691 (104.6)  **Postoperative complications (other than pancreatic fistula):** RR 0.91 (95% CI: 0.67, 1.24)  **Postoperative pancreatic fistula:** RR 1.20 (95% CI: 0.71, 2.04)  **Reoperation:** RR 0.50 (95% CI: 0.11, 2.25)  **Length of stay (Days):** 9 (7.05) vs. 11 (5.5)  **Readmissions:** RR 1.05 (95% CI: 0.55, 1.98)  **90-Day postoperative mortality:** RR 0.67 (0.11, 6.23)  **Long-term mortality:** RR 0.43 (95% CI: 0.13, 1.41)  **Endocrine insufficiency:** RR 0.27 (95%CI: 0.06, 1.12)  **Exocrine insufficiency:** RR 0.13 (95%CI: 0.02, 0.97) |
| Cherif et al. 2012 [30] | Retrospective study | France | Mean age was 55 years; 67% were Females; 64% had Tumor location in Body; Tumor size 15mm | Enucleation vs. Central pancreatectomy | 67 (Enucleation: 45 vs. Central pancreatectomy: 22) | *Median follow up 37 months for Enucleation and 34 months for Central pancreatectomy*  **Mean (SD), Operative time (min):** 180 (10) vs. 240 (6.6)  **Mean (SD), Blood loss (mL):** 50 (25) vs. 300 (41.6)  **Postoperative complications (other than pancreatic fistula):** RR 0.97 (95% CI: 0.74, 1.29)  **Post operative pancreatic fistula:** RR 1.01 (95% CI: 0.71, 1.43)  **Reoperation:** RR 2.44 (95% CI: 0.30, 19.67)  **Mean (SD), Length of stay (Days):** 21 (3) vs. 21.5 (2.6)  **Mortality:** RR 0.49 (95% CI: 0.03, 7.45)  **Endocrine insufficiency:** RR 0.28 (95% CI: 0.07, 1.13)  **Exocrine insufficiency:** RR 0.24 (95% CI: 0.02, 2.55) |
| Talamini MA et al (1998) [31] | Retrospective study | USA | Mean age was above 50 years; Around 50% were Females; Around 50% had tumor in body; Average Size of tumor: <40mm | Enucleation vs. Resection | 36 (Enucleation: 10; Resection: 26) | *Mean follow up 42.9 months for Enucleation and 48.6 months for Resection*  **Mean (SD), Operative time (min):** 272 (42) vs. 313 (36)  **Mean (SD), Blood loss (mL):** 209 (35) vs. 511 (111)  **Postoperative pancreatic fistula:** RR 4.33 (95% CI: 1.26, 14.84)  **Mean (SD), Length of stay (Days):** 19.5 (7.05) vs. 10 (5.5)  **Mortality:** RR 0.22 (95% CI: 0.01, 3.70) |
| Zhang T et al (2012) [32] | Retrospective study | China | Mean age was above 55 years; Majority of subjects were females; Majority (>53%) had tumor in body/tail; Average Size of tumor: <30 mm | Enucleation vs. Distal pancreatectomy | 147 (Enucleation: 129; Distal pancreatectomy; 18) | *Mean follow up 45 months*  **Postoperative pancreatic fistula**: RR 0.41 (95% CI: 0.09, 1.91) |
| Luo Y et al (2009) [33] | Retrospective study | China | Median age: 42.7 years; 58.6% were Females; Tumor location 48% in Body; Tumors ranged from 11 to 36 mm (mean, 19 mm) | Enucleation vs. Distal pancreatectomy | 26 (Enucleation: 16; Distal pancreatectomy: 10) | **Mean (SD), Operative time (min):** 85 (22.5) vs. 174 (43.3)  **Mean (SD), Blood loss (mL)**: 230 (81.6) vs. 330 (150)  **Postoperative pancreatic fistula**: RR 5.8 (95% CI: 0.34, 97.86) |
