# Supplementary material for: Comparison of Outcomes of Enucleation vs. Standard Surgical Resection for Pancreatic Neoplasms: A Systematic Review and Meta-Analysis
Source: Front Surg. 2022 Jan 26;8:744316. doi: 10.3389/fsurg.2021.744316 (PMC8825491; doi:10.3389/fsurg.2021.744316)
Supplement: Supplementary file 2 [file Table_2.docx]

**Supplementary table 2. Search strategy for identification of studies to be included in the review**

| **Search strategy**  #1 (enucleation OR pancreatectomy OR surgical resection OR pancreaticoduodenectomy)  #2 (pancreatic neoplasm OR pancreatic malignancy OR pancreatic neuroendocrine tumour)  #3 (mortality OR death OR recurrence OR complication OR readmission OR treatment outcome)  #4 (#1 AND #2 AND #3)  #5 (Addresses[ptyp] OR Autobiography[ptyp] OR Bibliography[ptyp] OR Biography[ptyp] OR pubmed books[filter] OR Case Reports[ptyp] OR Congresses[ptyp] OR Consensus Development Conference[ptyp] OR Directory[ptyp] OR Duplicate Publication[ptyp] OR Editorial[ptyp] OR Systematic reviews OR Meta analysis OR Festschrift[ptyp] OR Guideline[ptyp] OR In Vitro[ptyp] OR Interview[ptyp] OR Lectures [ptyp] OR Legal Cases[ptyp] OR News[ptyp] OR Newspaper Article[ptyp] OR Personal Narratives [ptyp] OR Portraits[ptyp] OR Retracted Publication[ ptyp] OR Twin Study[ptyp] OR Video-Audio Media[ptyp])  #6 (#4 NOT #5) |
| --- |
