# Supplementary material for: Comparison of Outcomes of Enucleation vs. Standard Surgical Resection for Pancreatic Neoplasms: A Systematic Review and Meta-Analysis
Source: Front Surg. 2022 Jan 26;8:744316. doi: 10.3389/fsurg.2021.744316 (PMC8825491; doi:10.3389/fsurg.2021.744316)
Supplement: Supplementary file 3 [file Table_3.docx]

**Supplementary table 3. Author’s judgements about study quality using the adapted Ottawa-Newcastle Risk of Bias Assessment tool**

|  | Balzano et al (2003) | Cruz et al (2007) | Hackert et al (2011) | Casadei et al (2010) | Cauley et al (2012) | Cherif et al (2012) | Talamini et al (1998) | Zhang et al (2012) | Luo et al (2009) |
| --- | --- | --- | --- | --- | --- | --- | --- | --- | --- |
| Representativeness/appropriateness of participant selection  Random or consecutive recruitment=Y  Convenience sample=N  Not reported or unclear | Y | Y | Y | Y | Y | Y | Y | Y | Y |
| Control for baseline differences in cohorts  Similarity of groups at baseline or adjustment in analyses=Y  No attempt to control or adjust=N  Not reported=NR | Y | Y | Y | Y | Y | Y | Y | Y | Y |
| Loss to follow-up  Explanation provided for loss of participants and/or intention to treat=Y  No explanation =N | Y | Y | N | Y | N | N | Y | Y | Y |
| Masking of exposure to outcomes assessor  Description of masking=Y  No masking or no description =N | Y | Y | Y | Y | Y | Y | Y | Y | Y |
| Ascertainment of condition  Description of ascertainment/diagnostic criteria=Y  No description or patient self-report=N | Y | Y | Y | Y | Y | N | Y | Y | Y |
| Documentation of other treatment modalities  Documentation=Y  No documentation=N | Y | Y | Y | Y | Y | Y | Y | Y | Y |
| Extent to which valid outcomes are described  Adequate description of outcome=Y  Insufficient detail regarding outcome or follow-up time=N | Y | Y | Y | Y | N | Y | Y | N | Y |
| Prespecification of harms, mode of harms collection  Description of a list of harms assessed or monitoring=Y  No such description or passive harms collection=N  No adverse events reported=NA | Y | N | Y | N | Y | N | Y | N | Y |
| Financial Conflict of interest (COI)  Funding source reported=Y  Funding source not reported=N | Y | Y | Y | N | Y | Y | Y | Y | Y |
